# Perspective: Balance Assessments in Progressive Supranuclear Palsy: Lessons Learned

**DOI:** 10.3389/fneur.2022.801291

**Published:** 2022-01-27

**Authors:** Marian L. Dale, Austin L. Prewitt, Graham R. Harker, Grace E. McBarron, Martina Mancini

**Affiliations:** ^1^Balance Disorders Laboratory, Department of Neurology, Oregon Health and Science University, Portland, OR, United States; ^2^Department of Physical Therapy, Columbia University Irving Medical Center, Vagelos College of Physicians and Surgeons, New York, NY, United States

**Keywords:** progressive supranuclear palsy, balance, posturography, wearable sensors, gait

## Abstract

Many studies have examined aspects of balance in progressive supranuclear palsy (PSP), but guidance on the feasibility of standardized objective balance assessments and balance scales in PSP is lacking. Balance tests commonly used in Parkinson's disease often cannot be easily administered or translated to PSP. Here we briefly review methodology in prior studies of balance in PSP; then we focus on feasibility by presenting our experience with objective balance assessment in PSP-Richardson syndrome and PSP-parkinsonism during a crossover rTMS intervention trial. We highlight lessons learned, safety considerations, and future approaches for objective balance assessment in PSP.

## Introduction

Many studies have examined aspects of balance in progressive supranuclear palsy (PSP) (1–12), but guidance on the feasibility of standardized objective balance assessments in PSP is lacking. Balance tasks commonly used in Parkinson's disease (PD) often cannot be administered in or directly translated to PSP, and the nine subtypes of probable and possible PSP (13) show various degrees of balance deficits. Here we briefly review methodology in prior studies of balance in PSP; then we focus on feasibility by presenting our experience with objective balance assessment in PSP-Richardson syndrome (PSP-RS) and PSP-parkinsonism (PSP-P) during a crossover rTMS intervention trial.

### Clinical Scales for Balance in PSP

Clinical scales are the most common method of balance assessment in PSP. The PSP Rating Scale (PSPRS) (14) is a general scale addressing PSP symptoms, activities of daily living, mentation, speech and swallow, eye movements, dexterity, and gait and balance. Out of a total of 100 scale points, 16 are devoted to gait and balance tasks on exam (arising from a chair, gait, postural stability, and sitting down). An additional history item asks about estimated fall frequency if the subject attempts to walk unaided, i.e., with no access to a walking aid, such as a walker. Because many subjects already require regular walking aid use at the time of testing, we find that this answer skews to the maximum item score and is thus less useful for tracking in longitudinal or intervention studies. The PSPRS exceeds at capturing the full spectrum of PSP symptoms, but lacks granularity to objectively investigate changes in balance. For example, the PSPRS-gait subscore does not correlate with total sway path on objective posturography (3). The motor section of the Movement Disorders Society Unified Parkinson's Disease Rating Scale (MDS-UPDRS) (15) is often used in studies that contrast PSP and PD but the MDS-UPDRS is weighted more heavily for tremor than is needed in PSP, lacks relevant postural control tasks of standing without using arms and controlled standing to sitting, and provides a less granular assessment of postural stability compared to the equivalent pull test task on the PSPRS. The Balance Evaluation System Test (BESTest) (16) and its shorter version (Mini-BESTest) (17) target different balance control systems so that specific rehabilitation approaches can be applied for different balance deficits. The BESTest was shortened based on a factor analysis to improve clinical utilization (17). The Mini-BESTest is a 14-item test scored on a 3-level ordinal scale assessing 4 aspects of balance: sensory integration, anticipatory postural adjustments, automatic postural responses, and dynamic balance during gait. Although both the BESTest and Mini-BESTest are highly sensitive tests of balance, certain items may be too difficult to perform in PSP (i.e., the lateral push and release, standing on foam with eyes closed, etc). For this reason, the Mini-BESTest has not been consistently applied or validated in PSP. The Berg balance scale (18), commonly used in stroke and geriatric balance studies, addresses fourteen easily-implemented balance tasks, but lacks reactive postural control tasks and uneven support surface items. It has a ceiling effect (19), and it is not validated in PSP.

### Review of Laboratory Measurement of Balance in PSP

Various technologies have been used to assess aspects of balance in PSP. Early studies (2) used the Sensory Organization Test (SOT) on the Neurocom Balance Manager System (Clackamas, OR) to assess sensory integration of postural control (20) by combining a moveable force plate platform with moveable surrounding walls (for platform and visual sway, respectively). Static force plate posturography tests sagittal and medio-lateral sway in PSP (3, 4, 6, 7, 9), and can examine limits of stability the maximum excursion or lean without taking a step or losing balance (5, 8). Dynamic force plate posturography records center of pressure (CoP) shifts after platform perturbations, such as forward translations and toes-up (backward) tilts, to assess motor control in PSP (5). Wearable sensors can examine a variety of movements on normal ground in PSP and overcome the restrictions of force plates. For example, triaxial accelerometers have measured gait acceleration and vertical displacement in PSP (10). Motion analysis systems combine force plates with patient markers and video tracking to capture a breadth of gait and balance tasks in PSP (11), including joint kinematics (12), and have demonstrated high inter-lab reliability (21), but come with significant drawbacks including high cost, time-consuming marker placement, lengthy pre-processing to assign each marker to its corresponding biomechanical model, followed by lengthy data processing and analysis (22).

## Our Experience with Objective Balance Testing in PSP

During our ongoing repetitive cerebellar controlled TMS crossover trial in PSP (NCT04468932), in which subjects receive multiple sessions of multi-modal balance testing, we have learned important lessons about feasibility in PSP. We focus on probable PSP-RS and PSP-P subtypes (13). We do not yet have experience with objective balance testing in other variants of PSP, such as PSP-speech and language. We are sharing our experience in order to encourage safe practices and facilitate more objective balance testing in PSP; this is not meant to be an exhaustive recommendation of procedures. To capture the known backward postural instability in PSP-RS, we focus on postural sway in the sagittal plane (see sections Dynamic Posturography on the Neurocom System and Selected Mini-BESTest Items, Two-Minute Walk Test, and a 360-Degrees Turning in Place With Opal Sensors below). We also collect sway in the medio-lateral plane as it is important for fall prevention, and we include perturbation tasks to challenge stability (see sections Dynamic Posturography on the Neurocom System and Selected Mini-BESTest Items, Two-Minute Walk Test, and a 360-Degrees Turning in Place With Opal Sensors below). Finally, our assessment captures straight walking and turning (see section Selected Mini-BESTest Items, Two-Minute Walk Test, and a 360-Degrees Turning in Place With Opal Sensors below) for overall clinical relevance, and because a subset of patients with PSP have freezing of gait.

**Figure 1:**
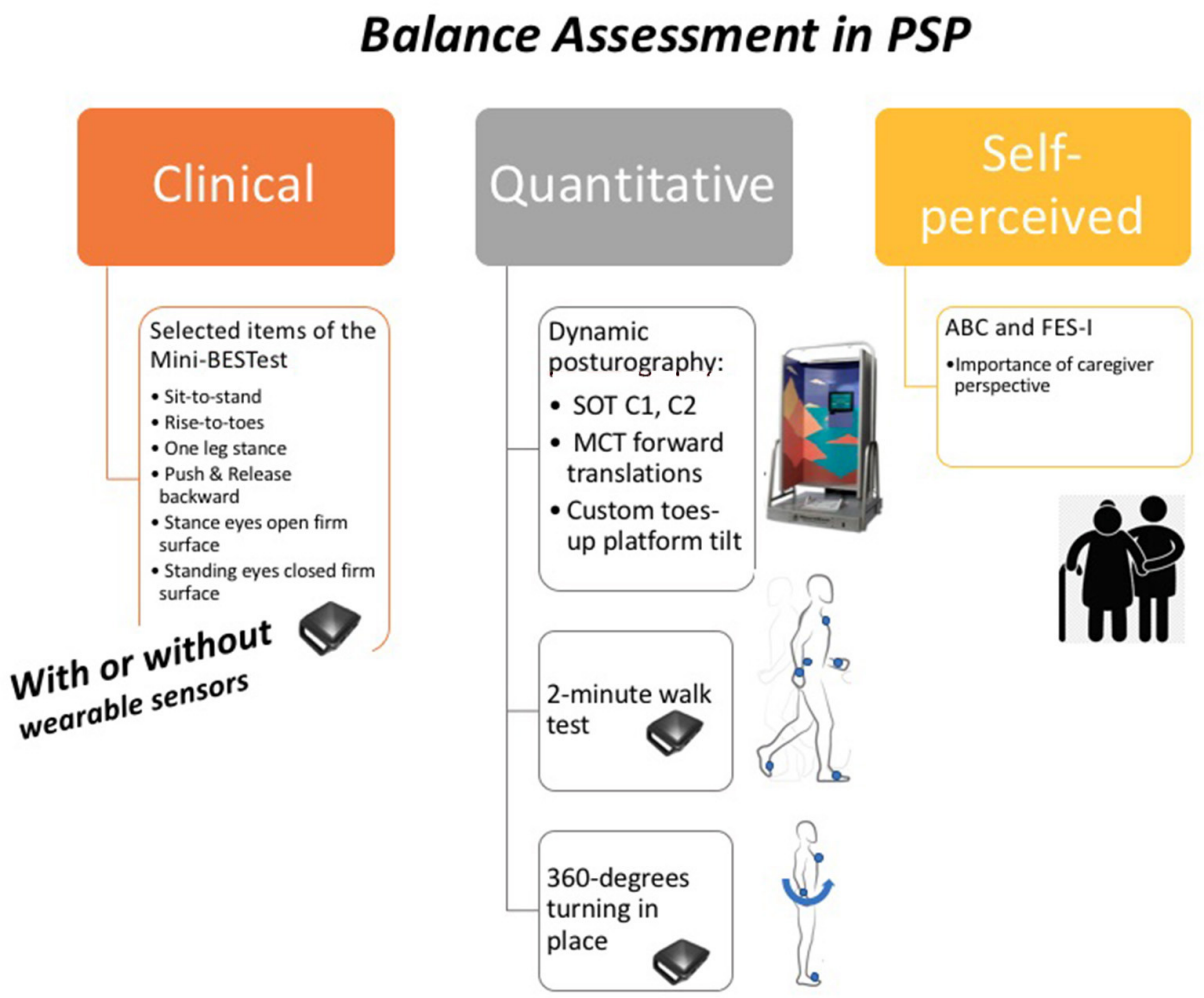
Our balance assessment in PSP protocol. SOT, Sensory Organization Test; C1, condition one (quiet stance without movement of the force plate or surround with eyes open); C2, condition two (quiet stance without movement of the force plate or surround with eyes closed); MCT, Motor Control Test; ABC, Activities-Specific Balance Confidence (ABC) Scale; FES-I, Falls Efficacy Scale-International.

Figure 1 shows our comprehensive balance assessment protocol for PSP: the Sensory Organization Test (SOT) and Motor Control Test (MCT) with forward platform translation and toes-up perturbations on a Neurocom Balance Manager system, anticipatory postural adjustments, reactive postural control and sensory orientation aspects of the mini-BESTest (17), a two-minute walk test (23, 24), and a 360-degree turning in place task (25). The mini-BESTest, two-minute walk, and 360-degree turning task are all performed while wearing six Opal inertial measurement sensors (APDM Wearable Technologies, Portland, OR) (26). We administer two balance quality of life questionnaires: the Activities-Specific Balance Confidence (ABC) Scale (27, 28) and Falls Efficacy Scale (FES-I) (29).

### Dynamic Posturography on the Neurocom System

We perform dynamic posturography on the Neurocom system to quantify sagittal and medio-lateral sway under various sensory conditions and with platform perturbations. The standard provided Neurocom output is an equilibrium score during each test, a sensory analysis score, and a strategy analysis (20). It is important to note that these outcomes purely rely on the sagittal sway during the tests, ignoring the medio-lateral sway. However, it is possible to download the force plate recording during the SOT tests and calculate both sagittal and medio-lateral COP excursion in all conditions. We also perform the large forward translations of the Motor Control Test (MCT). We include a customized toes-up platform tilting task because we previously found it differentiated subjects with PSP from PIGD-matched PD (5). Safety is ensured by a lightweight harness and an assistant for spotting. Trials are invalidated if subjects shift their feet on the surface of the force plate.

#### Feasibility

**Figure 2:**
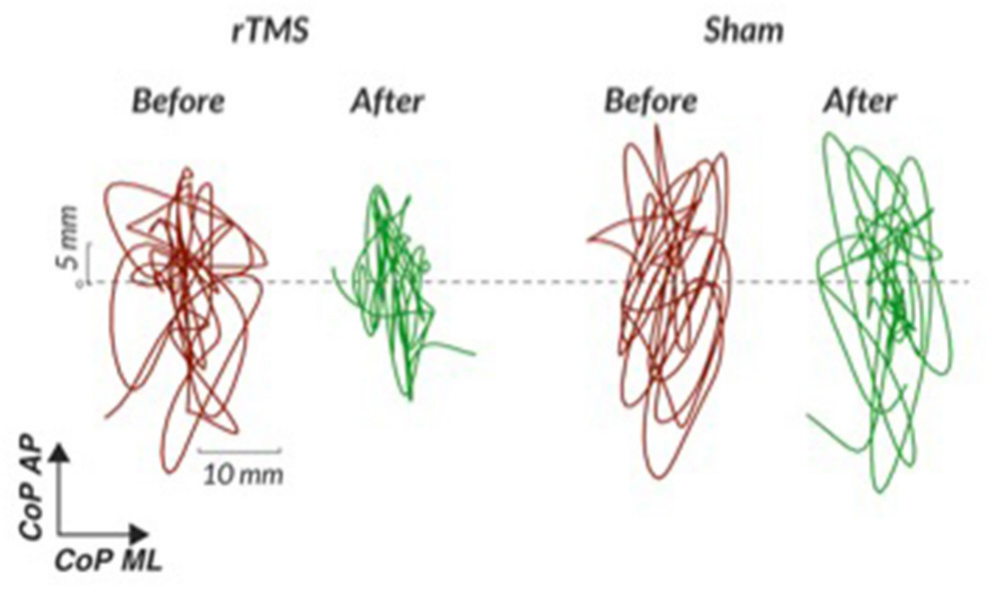
Representative center of pressure sway excursions in quiet stance without movement of the force plate or surround (condition one of the Sensory Organization Test) before and after cerebellar repetitive TMS compared to sham TMS. rTMS, repetitive transcranial magnetic stimulation; CoP, center of pressure; AP, anterior-posterior; ML, medio-lateral; C1, condition one.

We learned that the conditions most consistently completed without foot shifting during the SOT in PSP are conditions one through three (quiet stance without movement of the force plate or visual surround with eyes open, quiet stance without movement of the force plate or visual surround with eyes closed, and stance *with* movement of the visual surround with eyes open). See Figure 2 with representative center of pressure sway excursions in condition one before and after cerebellar repetitive TMS compared to sham TMS. The other elements of the SOT that involve force plate movement with or without eyes closed are generally challenging in our PSP subjects, though some subjects have shown individual improvements after our intervention. For example, 50% of our subjects were able to complete a condition of the SOT after rTMS that they could not complete without falls before rTMS, regardless of order of intervention. These individual improvements were not seen after sham TMS. For this reason, we suggest at least attempting to complete all aspects of the SOT, particularly in less impaired individuals. Our PSP subjects have generally tolerated perturbations with forward platform translations of the MCT and with toes-up platform tilts. While they may shift their feet during these perturbations and invalidate certain trials, a majority of trials are successfully completed and yield analyzable data. We find that the duration of posturography testing on the Neurocom system for more impaired subjects with PSP is 30 min, but the time becomes considerably shorter for less impaired subjects who are able to transfer in and out of the machine more efficiently.

#### Lessons Learned

1. Eye mask. It is necessary to use a comfortable eye mask to blindfold subjects for the eyes-closed portions of assessment, since abnormal eyelid function (caused by conditions such as apraxia of eyelid closing) can impair consistent eye closure in PSP. Subjects may not be able to close their eyes on command.
2. Standardized foot placement is essential. We recommend marking optimized foot placement on the force plate with tape. Geriatric neurological subjects may have concomitant chronic orthopedic issues (such as foot eversion) that prevent perfect alignment, so consistency during and between testing sessions is the goal.
3. Ensure subjects are consistently tested without footwear or socks, and either exclude or account for significant lower extremity proprioceptive deficits, such as loss of toe proprioception on neurological examination, in the study design.
4. Ensure that safety harness straps have some slack. Subjects with PSP often lean forward during testing to compensate for their backward postural instability. When leaning they may place sufficient tension on the harness straps to provide sensory input and mechanical support that invalidates posturography results.
5. Spotting during balance testing and assistance entering and exiting the Neurocom are essential for safety; the safety harness is necessary, but not sufficient. While the harness prevents full falls, subjects with PSP risk injuring themselves on the walls of the Neurocom during perturbations. Subjects often need assistance stepping into and out of the machine.
6. Clearly marking “falls” or foot shifting that invalidate trials in the study case report form assists in data analysis.

### Selected Mini-Bestest Items, Two-Minute Walk Test, and a 360-Degrees Turning in Place With Opal Sensors

Compared to posturography confined to the Neurocom machine, these tests are more representative of real-life challenges to static and dynamic balance. For this mobile testing we equip patients with six lightweight Opal sensors (APDM Wearable Technologies, Portland, OR) (26) (one on each wrist, one on each ankle, one on the chest and one over the lumbar area with Velcro straps). The inertial sensors combine accelerometer, gyroscope, and magnetometer technology along three axes. We then “instrument” the mini-BESTest by performing it while subjects wear the mobile sensors. The full mini-BESTest is a fourteen-task scale addressing anticipatory postural adjustments, reactive postural control, sensory orientation, and dynamic gait. We perform portions of the mini-BESTest with Opal sensors in PSP as noted in the Feasibility section below. Then, in the 2-min unassisted walk, subjects walk uninterrupted with mobile sensors back and forth down a hallway. Spatio-temporal gait characteristics, such as stride length, gait speed, angle of the foot at heel-strike, and upper body arm swing and trunk angle while walking are calculated from the 2-minute walk test (26, 30). Both average and variability are reported. For the separate instrumented 360 degrees turning in place task, subjects are instructed to turn in place for a total of 1 min, 360 degrees to the right, then 360 degrees to the left (and so on) at a comfortable speed (25). This turning protocol elicits potential freezing of gait in a controlled manner.

#### Feasibility

Thus, far in six subjects with PSP (each with multiple testing sessions), we find that subjects diagnosed with probable PSP-RS or PSP-P are unable to complete all portions of the mini-BESTest without adjustments that invalidate results. We suggest limiting mini-BESTest tasks to the following: sit-to-stand, rise to toes, stand on one leg, compensatory stepping correction backward, stance with eyes open on a firm surface, and stance with eyes closed on a firm surface. All six tasks will not be feasible in all patients, but all are worth attempting. In our experience, even with two highly trained assistants per subject for safety spotting, the following mini-BESTest tasks are generally not feasible and may be eliminated: compensatory stepping correction forward, compensatory stepping correction lateral, stance with eyes closed on a foam surface, and stance with eyes closed on an incline. We have been surprised that compensatory stepping correction backward is more feasible than compensatory stepping forward in PSP, but this mainly relates to reluctance of subjects to sufficiently transfer their weight to the examiner at the beginning of the forward compensatory stepping task, invalidating any results. We find the dynamic gait portion of the mini-BESTest, which includes items such as straight walking with head version, too difficult in PSP; instead, we recommend incorporating mobile sensor testing into separate 2-min unassisted walking and 360 degree turn tasks to obtain quantitative spatio-temporal parameters of gait and turning. The average duration to complete the instrumented mini-BESTest items, the 360 degrees turning in place task, and the 2-min walk test is 45 min.

#### Lessons Learned

1. We modified instructions for selected tests of the mini-BESTest to account for the wider base of balance often necessary in PSP, even in less advanced subjects. For example, during the eyes open standing on a firm surface test we use a template to maintain a consistent distance between the feet at different sessions, as opposed to a variable patient-selected stance width. The original mini-BESTest instructions of standing with feet nearly touching is often not feasible in this population. We first try standing with eyes open using a template between the feet. If subjects are able to complete this task, we then add the more challenging task of standing on a firm surface with eyes closed and feet together.
2. Consistency in subject testing with shoes and socks off is important for validity.
3. Two spotters are often required for all mobile sensor testing in order to safely push most subjects to the limits of their balance capabilities. A gait belt is required.
4. Monitor for impulsivity during the unassisted gait test. Certain patients with PSP may walk quickly and precariously with a high initial acceleration (10). We caution subjects to “walk at your normal pace; you do not have to rush,” rather than instructing them to walk as quickly as they can. We are more interested in quality metrics such as gait variability than total distance covered.
5. During unassisted gait, some subjects with PSP may move their head more than a healthy age matched control in an to attempt to overcome their oculomotor deficits and visually scan their surroundings. This can distract subjects from the task. If this behavior occurs, we gently correct and remind subjects to keep looking straight ahead during the gait testing.

### Patient-Rated Balance Questionnaires

We collect the Activities-Specific Balance Confidence (ABC) Scale (27, 28) and Falls Efficacy Scale-International (FES-I) (29) questionnaires from both the subject and caregiver. We have not seen improvement in either the ABC or FES-I that corresponds to static posturography improvements. This could either mean that static postural tasks do not capture clinically relevant and dynamic balance skills, *or* that questionnaires are not sensitive enough to detect objective instrumented improvements that would continue to improve with a longer intervention or training. Future longitudinal studies are needed.

#### Lessons Learned

1. We find that subjects may overestimate their balance abilities, particularly in intervention trials, so it is important to separately collect the caregiver perspective.
2. ABC and FES-I scales are scored in opposite directions, such that a 100% on the ABC represents total confidence in one's balance abilities, while a high score on the FES-I represents low confidence that one could do various activities without falling. Due to executive dysfunction and perseveration in PSP (31), certain subjects become confused and report answers that are the opposite of their intended answers. It is important to remind subjects of the instructions, to consider using only one scale, or to separate administration of the scales with other study tasks.
3. The average time for caregivers to complete the ABC and FES-I scale is 10 min. The subjects themselves may take up to 20 min to complete the scales with examiner assistance due to (1) bradyphrenia and (2) speech impairments that require them to repeat themselves or to point to answers for interpretability.

### General Safety Considerations and Patient Comfort

Consideration of fall prevention at every point of contact in studies of PSP is paramount. The study team must consider fall prevention during patient transport to and from their vehicle, while navigating large research facilities, during bathroom breaks, in the MRI suite, etc. As caregivers know, this is not a trivial task. We recommend transporting patients in a wheelchair to and from their vehicle as well as while navigating the research facility. Normalizing wheelchair transport as a standard study procedure improves safety and prevents excessive subject fatigue, an important benefit because fatigue may confound balance testing results. It is important to be mindful to test subjects at consistent times of the day to minimize confounding affects related to alertness level. Because a subset of subjects with PSP may be on levodopa, ensuring consistent assessment times related to medication administration times is essential, especially since levodopa can increase postural sway (32). During testing and transport we recommend constant use of a lightweight gait belt without metal parts. In the case of a study with a MR imaging component, gait belts without metal fasteners can safely enter the MR suite without last minute awkward reconfigurations. It is imperative that MR technicians be trained in fall risk in PSP, and it is additionally recommended that research assistants are present in the MR suite and available to assist the MR tech with patient transfer in and out of the scanner. Regarding patient comfort during testing, we find that most patients prefer on-ground testing with mobile sensors and two spotters to being in the Neurocom with a harness and one spotter.

## Future Directions

Alternative methodologies may better target balance deficits in PSP in the future. Dynamic posturography will benefit from force plates with seated testing capabilities, such as the Hunova system (Movendo Technologies, https://www.movendo.technology/en/) (33, 34). Seated assessment will be especially beneficial for more advanced subjects and for sit-to-stand training. The ZeroG Gait and Balance system (Aretech llc, https://www.aretechllc.com/) is a dynamic body-weight support system that has the potential to increase the safety of targeted rehabilitation programs for postural instability in PSP. Video motion analysis systems capture a breadth of movement tasks with high reliability (21), but we believe that inertial sensors and marker-less technologies reduce data processing time systems with similar accuracy and without the need for trained personnel for pre-processing (22). Turning is especially difficult to measure in video motion analysis systems because markers can become obstructed during transitions unless special measures are implemented (22). Intricate lab-based video motion analysis systems will not transition as easily as mobile sensors to home based or telehealth assessments in future clinical trials.

## Conclusion

Balance testing in PSP is quickly moving beyond scale-based ratings to more objective assessments. Objective assessments in PSP should ideally capture multiple aspects of balance, including static balance, gait, turning, joint kinematics, and cognitive aspects of mobility. Safety can be ensured by consistent implementation of careful protocols by trained teams of neurologists, PTs, and study personnel familiar with PSP. Data integrity in future multi-center trials of balance in PSP will depend on consistent methodologies and patient instructions. Future studies are needed to examine balance deficits in the less common subtypes of PSP, and recruitment in early PSP is essential.

## Data Availability Statement

The raw data supporting the conclusions of this article will be made available by the authors, without undue reservation.

## Ethics Statement

The studies involving human participants were reviewed and approved by Oregon Health and Science University. The patients/participants provided their written informed consent to participate in this study.

## Author Contributions

MD: conception, data gathering, and writing. AP, GH, and GM: data gathering and reviewing. MM: organization, writing, and reviewing. All authors contributed to the article and approved the submitted version.

## Funding

The project described was supported by the National Center for Advancing Translational Sciences, National Institutes of Health, through Grant (No. KL2TR002370 to MD). The authors would also like to acknowledge the support of the NIH NC-NM4R Pilot Project Grant (No. P2CHD086844 to MD), the Collins Medical Trust to MD, and the NIH (No. R01-HD100383 to MM).

## Author Disclaimer

The content is solely the responsibility of the authors and does not necessarily represent the official views of the NIH.

## Conflict of Interest

The authors declare that the research was conducted in the absence of any commercial or financial relationships that could be construed as a potential conflict of interest.

## Publisher's Note

All claims expressed in this article are solely those of the authors and do not necessarily represent those of their affiliated organizations, or those of the publisher, the editors and the reviewers. Any product that may be evaluated in this article, or claim that may be made by its manufacturer, is not guaranteed or endorsed by the publisher.
