# Supplementary material for: Perspective: Balance Assessments in Progressive Supranuclear Palsy: Lessons Learned
Source: Front Neurol. 2022 Jan 27;13:801291. doi: 10.3389/fneur.2022.801291 (PMC8828584; doi:10.3389/fneur.2022.801291)
Supplement: Supplementary file 1 [file Table_1.DOCX]

Supplementary Table 1. Participant Demographics

| Participant | Gender | Age | PSPRS | MoCA |
| --- | --- | --- | --- | --- |
| 1 | F | 65 | 45 | 13 |
| 2 | M | 77 | 33 | 24 |
| 3 | M | 71 | 33 | 24 |
| 4 | F | 83 | 43 | 13 |
| 5 | M | 72 | 53 | 20 |
| 6 | M | 66 | 58 | 15 |

PSPRS= PSP Rating Scale (out of total score of 100,

lower scores are better); MOCA= Montreal Cognitive

Assessment (out of total score of 30, higher scores

are better)


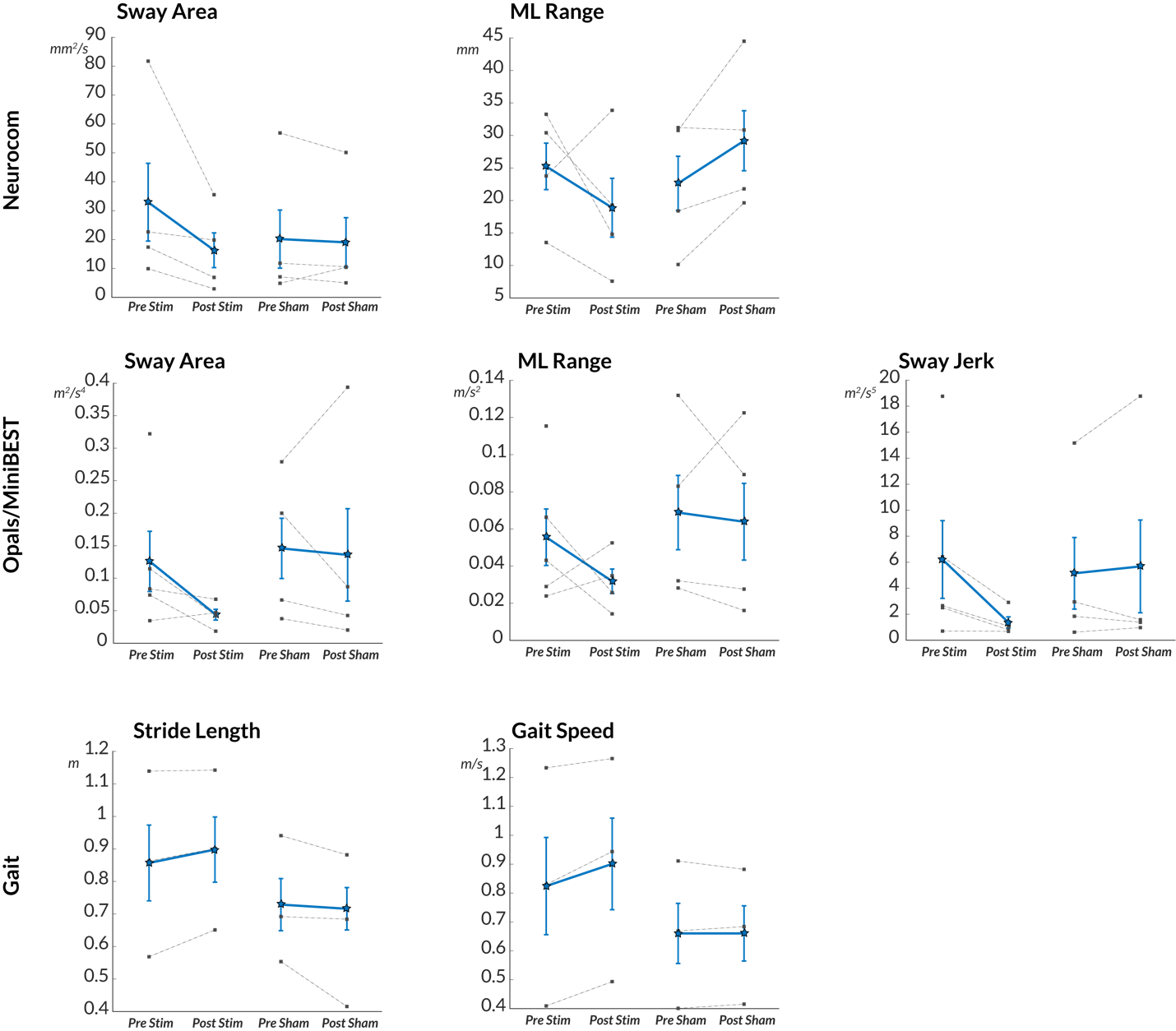
 Supplementary Figure 1. Objective balance and gait outcomes from: 1) Neurocom, condition 1 of the SOT, Sway Area and medio-lateral (ML) range calculated from the center of pressure; 2)

Sway area, medio-lateral range and sway jerkiness from 30s of standing with eyes open for one item of the MiniBESTest; 3) Stride length and gait speed during a 2-minute walk test computed from Mobility Lab (APDM Wearable Technologies, v2). All 6 subjects did not complete all tasks in this preliminary protocol. Statistical analysis is pending larger sample size. The Neurocom motor control tests and 360 degrees turning data have not yet been analyzed.
